# GFPT2 promotes paclitaxel resistance in epithelial ovarian cancer cells via activating NF-κB signaling pathway

**DOI:** 10.1515/biol-2022-1039

**Published:** 2025-04-24

**Authors:** Zi-Jun Xu, Bin Liu, Ruo-Nan Li, Hua Linghu

**Affiliations:** Department of Gynecology, The First Affiliated Hospital of Chongqing Medical University, Chongqing, 400016, China; Department of Pathology, The Basic Medical School of Chongqing Medical University, Chongqing, 400016, China

**Keywords:** GFPT2, paclitaxel, chemotherapy resistance, epithelial ovarian cancer, apoptosis, NF-κB

## Abstract

This study investigated the role of glutamine-fructose-6-phosphate transaminase 2 (GFPT2) in the response of epithelial ovarian cancer cells to paclitaxel, a standard chemotherapy drug. We analyzed GFPT2 expression across various EOC cell lines, including SKOV3, HEY, ES-2, A2780, and OVCR3. In HEY cell lines, we performed GFPT2 knockdown, while A2780 cells were engineered for GFPT2 overexpression. Following these manipulations, we assessed the cellular responses to paclitaxel treatment. Results demonstrated a correlation between GFPT2 levels and paclitaxel resistance; those with high GFPT2 (SKOV3 and HEY) expression were less sensitive compared to the cells with low GFPT2 expression (A2780). Downregulating GFPT2 enhanced drug sensitivity in HEY cells, whereas its overexpression impaired drug sensitivity in A2780 cells. Mechanistically, GFPT2’s role in facilitating paclitaxel resistance was linked to the activation of the nuclear factor-κB (NF-κB) signaling pathway, possibly influenced by NK3 Homeobox 2. Our findings suggest that GFPT2 is a critical mediator of paclitaxel resistance through NF-κB pathway activation in EOC, providing potential targets for improving therapeutic efficacy against this challenging malignancy.

## 1 Introduction

Epithelial ovarian cancer (EOC) ranks among the most aggressive malignancies affecting the female reproductive system [1]. Its insidious onset and absence of prominent clinical indicators often lead to advanced-stage diagnosis [1]. The inadequacy of early diagnostic methods further contributes to the disheartening 5-year survival rates associated with this disease [1]. The likelihood of developing EOC rises with age, particularly affecting women between 50 and 70 [1]. Over 70% of ovarian cancer patients are diagnosed at advanced stages (Federation International of Gynecology and Obstetrics stage III/IV), where the chances of optimal surgical resection fall below 50% [1]. Consequently, chemotherapy regimens incorporating paclitaxel become crucial for improving the survival rates of patients with EOC [2]. Unfortunately, chemoresistance poses a significant challenge, significantly hindering treatment effectiveness and contributing to cancer recurrence [2]. Alarmingly, more than 80% of patients with EOC experience a relapse within 24 months of initial treatment [2].

Paclitaxel has been a standard therapeutic agent for EOC [2]. Nevertheless, the development of drug resistance and insensitivity still hinders its therapeutic effect [2]. Investigations on the mechanism of action of paclitaxel have demonstrated that it promotes chromosomal instability in cancer cells and thus elicits its cytotoxic effect [3]. Even with this action, therapy resistance is developed by several high-grade serous ovarian cancers (HGSOC), which comprise more than seventy percent of EOC cases and are prone to chromosomal instability [4]. Therefore, understanding various pathways of paclitaxel resistance mechanisms is still lacking in practice. Thus, it is essential to investigate alternative target pathways capable of overcoming this resistance.

Glutamine-fructose-6-phosphate transaminase 2 (GFPT2) is known to be the first and rate-limiting enzyme of the hexosamine biosynthesis pathway (HBP) [5]. This enzyme is critical in several cellular activities, particularly glycosylation events [5]. GFPT2 is quite frequently overexpressed in several carcinomas, which might suggest its importance in cancer metastasis. Our earlier work illustrated that GFPT2 promotes epithelial–mesenchymal transition (EMT) in HGSOC cells through β-catenin nuclear localization [6]. Building upon previous research, the current study aims to elucidate the connection between GFPT2 expression levels and the resistance of EOC cells to paclitaxel treatment. Our findings reveal a previously unexplored role of GFPT2 in modulating paclitaxel resistance through activation of the nuclear factor-κB (NF-κB) signaling pathway. Furthermore, increased GFPT2 expression correlates with adverse treatment outcomes in EOC patients receiving paclitaxel therapy. This highlights GFPT2’s potential as a critical factor in the battle against EOC chemoresistance, suggesting avenues for improved therapeutic strategies.

## 2 Materials and methods

### 2.1 Cell culture

The HEY cell line was generously provided by Shanghai Jikai Gene Chemical Technology. The SKOV3 cell line was obtained from the American Type Culture Collection. The OVCAR3, ES-2, A2780, and ISOE cell lines were acquired from the cell bank of the Chinese Academy of Sciences, Shanghai. RPMI-1640 medium containing 10% fetal bovine serum (FBS) and 1% penicillin/streptomycin was used to maintain all cell lines. The culture conditions were 37°C at 5% CO_2_ in an incubator.

### 2.2 Lentivirus packaging and transfection

Lentiviral vectors expressing full-length GFPT2 expression were synthesized by Shanghai Jikai Gene Chemical Technology. Lentiviral vectors carrying GFPT2 shRNA were provided and synthesized by Gemma Company. The cells were seeded for 24 h in six-well plates containing (approximately 5  ×  10^4^ cells/well). Lentivirus was incorporated into the cells the next day (multiplicity of infection = 80), and the cells were incubated at 37°C in RPMI-1640 culture medium without serum. Cells were then maintained according to standard procedures, which included culturing in RPMI-1640 medium with 10% FBS after 24 h. After growing for 72 h, cells that were stably knocked down and over-expressed the *GFPT2* gene were selected using 5 µg/ml of puromycin. The selection period lasted approximately 4 days until no significant cell death was observed.

### 2.3 CCK-8 cell viability assay

Cells in the logarithmic growth phase were resuspended, counted, and seeded into 96-well plates at a density of 5,000 cells per well. Each well received a 100 µl aliquot of culture medium. Paclitaxel was added in varying concentrations to achieve final levels of 0, 5, 10, 15, 20, 40, 60, and 80 nM, respectively. After 72 h of incubation at 37°C with 5% CO_2_, 10 µl of cell counting kit-8 (CCK-8) solution was added to each well and incubated for 1 h. Following this incubation, the optical density was measured at 450 nm using a microplate reader, allowing the calculation of cell viability rates.

### 2.4 RNA extraction and real-time quantitative reverse transcription polymerase chain reaction

When the cell density reached 70–80%, TRIzol reagent (Takara, Tokyo, Japan) was used to extract total RNA. Following extraction, the RNA underwent reverse transcription into cDNA with a reverse transcription kit (Prime Script™ RT reagent Kit with gDNA Eraser), following the manufacturer’s guidelines. Real-time PCR was conducted using SYBR and the appropriately mixed primers. The cycling conditions consisted of an initial denaturation at 95°C for 5 min, followed by 40 cycles at 95°C for 10 s and 60°C for 30 s, concluding with a final step at 65°C for 5 s. The comparative CT method was employed to normalize the data, which was subsequently analyzed. The primers used in the study were as follows: *GAPDH* (sense primer: 5′-TTTGTGATGGGTGTGAACCACG-3′; anti-sense primer: 5′-TTGTGAGGGAGATGCTCAGTGTTG-3′), *NKX3-2* (sense primer: 5′-TTCCAGAACCGTCGCTACAAG-3′; anti-sense primer: 5′-CAGGTATTGTCTCTGGTCGTCG-3′), and *GFPT2* (sense primer: 5′- TCGCCAAATGCCAGAACG-3′; anti-sense primer: 5′-GCAAACTTGGAACTTTCAGTATCG-3′).

### 2.5 Western blot assay and related antibodies

Once cell density reached 70–80%, radioimmunoprecipitation assay (RIPA) lysate with 1% phenylmethanesulfonyl fluoride and 0.1% phosphatase inhibitor was used to extract the total protein. The bicinchoninic acid protein assay kit determined protein concentrations, with absorbance measured at 562 nm using a microplate reader, before proteins were stored at −80°C. Protein samples (30–50 μg) were separated using 8, 10, or 12% sodium dodecyl sulfate-polyacrylamide gel electrophoresis gels and transferred to polyvinylidene fluoride membranes. Overnight incubation at 4°C with primary antibodies was followed by a secondary antibody incubation for 1 h at room temperature after tris-buffered saline and tween 20 (TBST) washing. Membranes were washed thrice for 30 min with TBST before bands were visualized with the efficient chemiluminescence kit (ECL) developer and analyzed using Fusion software. Reagents, including RIPA lysis buffer, inhibitors, and secondary antibodies, were from Shanghai Beyotime Biotechnology, and the ECL developer was from Wanlei, China. Primary antibodies for GFPT2 were from Abcam (UK), while those for p65, GAPDH, NKX3-2, and Caspase-3 were from Proteintech Biotechnology (Wuhan, China), and P-p65 from Cell Signaling Technology (CST, USA), with dilutions as follows: GFPT2 1:1,500, NKX3-2 1:300, GAPDH 1:1,000, phosphorylated p65 (P-p65) 1:1,000, total p65 (T-p65) 1:1,000, and cleaved Caspase-3 1:1,000.

### 2.6 Immunofluorescence

During the logarithmic growth phase, we transferred cells, counted their numbers, and seeded them evenly across cell climbing dishes set within a 24-well format, aiming for a uniform distribution of 50,000 cells per well. After a 48-h sojourn in a nourishing medium, the cells underwent a stabilizing process with a 4% paraformaldehyde solution for 20 min, which was succeeded by a triple rinse with PBS to cleanse the cells. A 0.5% Triton X-100 solution was meticulously added to facilitate cell membrane permeation. Following this, the cells were blocked with goat serum for 2 h, and without any intervening wash, they were then immersed in a cold incubation chamber at 4°C overnight with a specific antibody targeting P-p65, diluted to a precise ratio of 1:100. The following day, in an environment shielded from light, cells were treated with goat anti-rabbit IgG secondary antibodies. Afterward, they were rinsed three times using PBS. The cells were then incubated with DAPI in darkness for 1 min. Following another wash with PBS, the cell climbing slides were removed from the 24-well plates and sealed with tablets designed to prevent fluorescence quenching. Finally, an anti-fade mounting medium was used to mount the coverslips. Images were captured using a laser confocal microscope. The DAPI used in the experiment was obtained from Shanghai Beyotime Biotechnology, while the Cy3-labeled goat anti-rabbit IgG (H + L) secondary antibody was sourced from Wuhan Proteintech Biotechnology.

### 2.7 Gene set enrichment analysis (GSEA)

Transcriptomic data on ovarian cancer patients were retrieved from The Cancer Genome Atlas (TCGA) database (https://portal.gdc.cancer.gov/). Data from patients exhibiting both high and low levels of GFPT2 expression were chosen, and enrichment analysis was carried out utilizing GSEA version 4.0.3.

### 2.8 Statistical analysis

Data analysis was performed using GraphPad Prism version 7.0. The results are depicted as the average ± standard deviation for continuous variables and were subjected to *t*-test analysis. Discrete data were assessed using the Chi-square test. A threshold for statistical significance was set at *P*-values below 0.05 (*P* < 0.05).

## 3 Results

### 3.1 Downregulation of GFPT2 expression increases the responsiveness of ovarian cancer cells to paclitaxel

**Figure 1:**
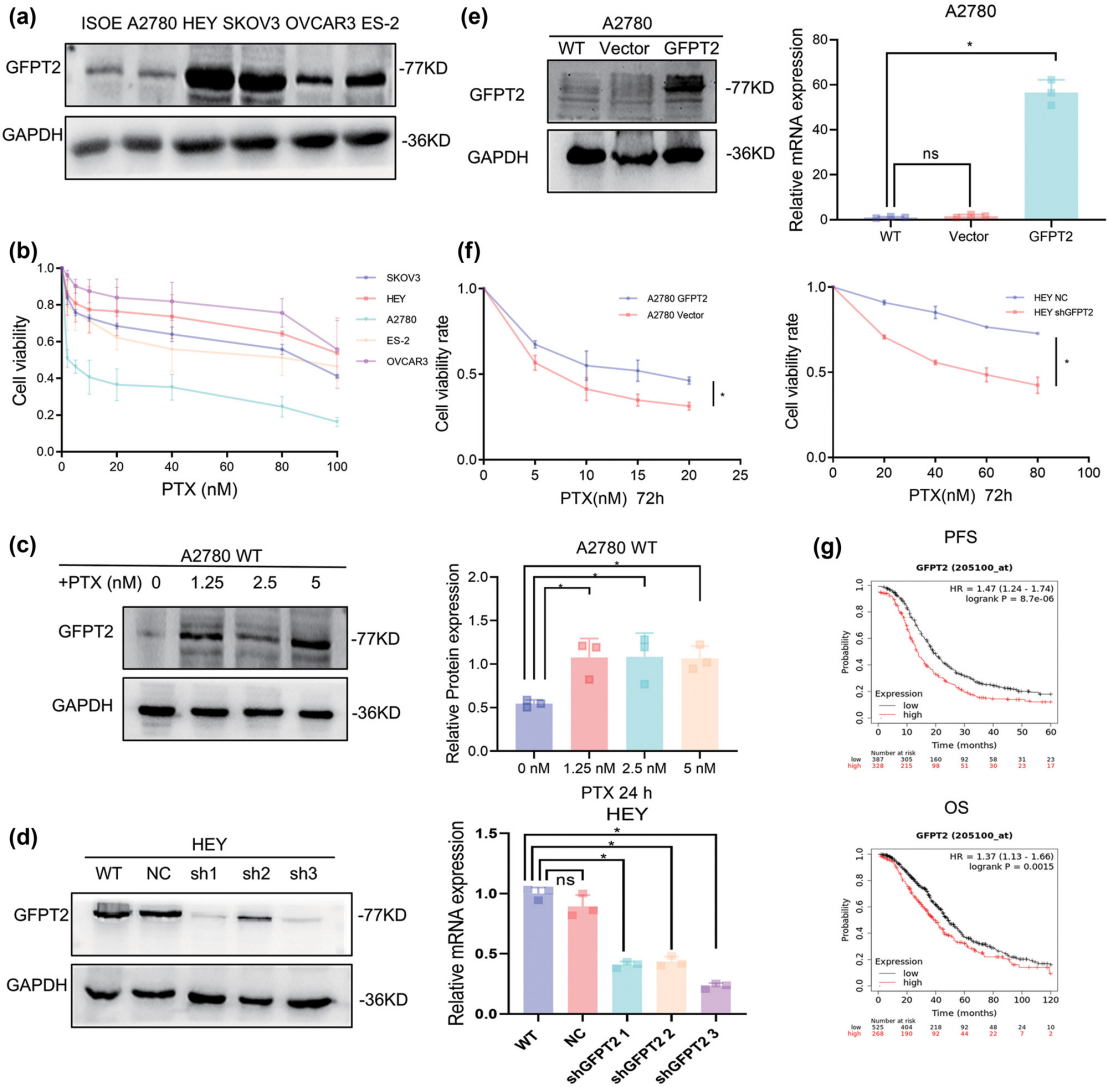
GFPT2 participates in the paclitaxel resistance in A2780 and HEY cells. (a) GFPT2 expression levels in various ovarian cancer cell lines; (b) ovarian cancer cells were treated with paclitaxel at concentrations of 2.5, 5, 10, 20, 40, 60, 80, and 100 nM, and cell viability was analyzed using the CCK-8 assay. (c) Wild-type A2780 cells were treated with various low concentrations of paclitaxel for 24 h, and GFPT2 expression was detected. (d) GFPT2 expression was knocked down in HEY cells, which exhibit high endogenous GFPT2 expression and resistance to paclitaxel. (e) GFPT2 was overexpressed in A2780 cells, which display low endogenous GFPT2 expression and a favorable response to paclitaxel. (f) HEY and A2780 cells were treated with a stepwise concentration of paclitaxel (A2780: 5, 10, 15, 20 nM; HEY: 20, 40, 60, 80 nM) for 72 h; cell viability was measured by CCK-8 assay, and the impact of GFPT2 expression on paclitaxel responsiveness was analyzed. (g) Progression-free survival and overall survival in patients with ovarian cancer who received Taxol-containing chemotherapy, as derived from TCGA database. Results are expressed as means ± standard deviation. *P < 0.05.

Attempting to investigate a relationship between GFPT2 and ovarian neoplasm, we performed experiments on several ovarian cancer cell lines, such as A2780, HEYA8, OVCAR3, ES-2, and SKOV3, in addition to one normal human ovarian cell line, IOSE80. Western blotting showed that the HEY cell line contains the highest, whereas the A2780 had lesser GFPT2 levels than all the other lines in a spectrum ranging from upper to lower expression of GFPT2 protein level (Figure 1a). These observations led us to select the HEY and A2780 lines for a more in-depth study. Among the cell lines, A2780, which featured minimal GFPT2 expression, was notably responsive to paclitaxel treatment, while the HEY line, along with others showing higher GFPT2 expression, displayed a significant level of resistance to the treatment (Figure 1b). To explore how GFPT2 expression might relate to resistance to paclitaxel, we subjected the A2780 line to a variety of low doses of paclitaxel for a 24-h period and noticed a subsequent rise in GFPT2 protein levels (Figure 1c). In our quest to further understand this relationship, we established stable cell lines with reduced GFPT2 expression through the use of shRNA lentiviral particles, with shRNA-3 being selected for our experiments that followed (Figure 1d). In parallel, we also created stable cell lines in A2780 that overexpressed GFPT2, setting the stage for a comparison with the original A2780 cells (Figure 1e). Our findings pointed to a connection between GFPT2 levels and sensitivity to paclitaxel; the reduction of GFPT2 in HEY cells heightened their sensitivity to paclitaxel, whereas an increase in GFPT2 in A2780 cells resulted in a greater degree of resistance (Figure 1f). To gain insight into the broader clinical implications of GFPT2 expression concerning the response to paclitaxel and survival rates in ovarian cancer patients, we reviewed data from individuals within TCGA database who underwent chemotherapy treatments containing paclitaxel. Using Kaplan–Meier survival methods, our analysis indicated that an elevated *GFPT2* expression was linked to a less favorable prognosis (Figure 1g). These results demonstrated that high GFPT2 expression is associated with decreased sensitivity to paclitaxel in ovarian cancer cells.

### 3.2 GFPT2 reduces paclitaxel sensitivity by inhibiting the paclitaxel-mediated pro-apoptotic effect in EOC cells

**Figure 2:**
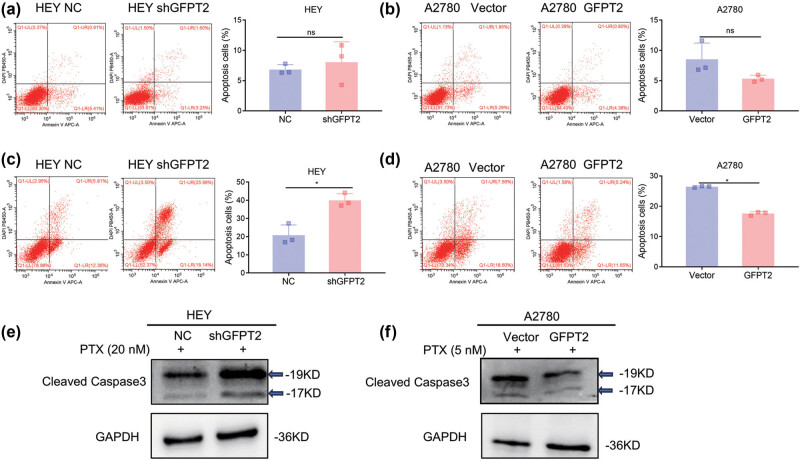
GFPT2 inhibits paclitaxel-induced apoptosis of A2780 and HEY cells lines. (a) Following staining with Annexin V APC-A/DAPI, apoptosis ratios in HEY cells with or without GFPT2 knockdown were determined by flow cytometry analysis. (b) Similarly, apoptosis ratios were analyzed in A2780 cells with or without forced GFPT2 expression. (c) and (d) After treatment with paclitaxel (A2780: 5 nM; HEY: 20 nM) for 72 h, apoptosis ratios in HEY and A2780 cells, with or without alterations in GFPT2 expression, were determined through Annexin V APC-A/DAPI double staining for Flow cytometry analysis. (e) and (f) Under the same treatment conditions, apoptosis was measured by cleaved Caspase-3 expression in HEY and A2780 cells, with GAPDH serving as a control. Results are expressed as means ± standard deviation. *P < 0.05.

To explore the potential role of GFPT2 in influencing the sensitivity of EOC cells to paclitaxel, our initial focus was on cell death, specifically apoptosis, in the context of GFPT2 overexpression. We conducted preliminary experiments without paclitaxel and observed that cells exhibiting higher levels of GFPT2 displayed a marginally lower rate of cell death. However, this variation was not statistically significant (Figure 2a and b). Following this, we examined how alterations in GFPT2 levels affected paclitaxel-induced apoptosis. We exposed different cell lines to varying drug concentrations over 3 days – specifically, A2780 cells were treated with 5 nM and HEY cells with 20 nM. The apoptosis assays indicated that decreasing GFPT2 in HEY cells heightened their sensitivity to paclitaxel (Figure 2c). Conversely, as shown in Figure 2d, elevating GFPT2 levels in A2780 cells led to a reduced sensitivity to the drug, reflected in the changes in apoptosis rates. We also employed western blot analysis to quantify the levels of cleaved Caspase-3, an established apoptosis marker, in ovarian cancer cell lines post-paclitaxel treatment. In HEY cells with diminished GFPT2 expression, we noted an increase in cleaved Caspase-3 levels following paclitaxel exposure compared to control cells (Figure 2e). In contrast, A2780 cells with heightened GFPT2 expression exhibited a decrease in cleaved Caspase-3 levels post-treatment relative to control cells (Figure 2f). These findings suggest that GFPT2 is instrumental in modulating the apoptotic response of ovarian cancer cells to paclitaxel.

### 3.3 GFPT2 modulates the sensitivity to paclitaxel by activating the NF-κB signaling pathway

**Figure 3:**
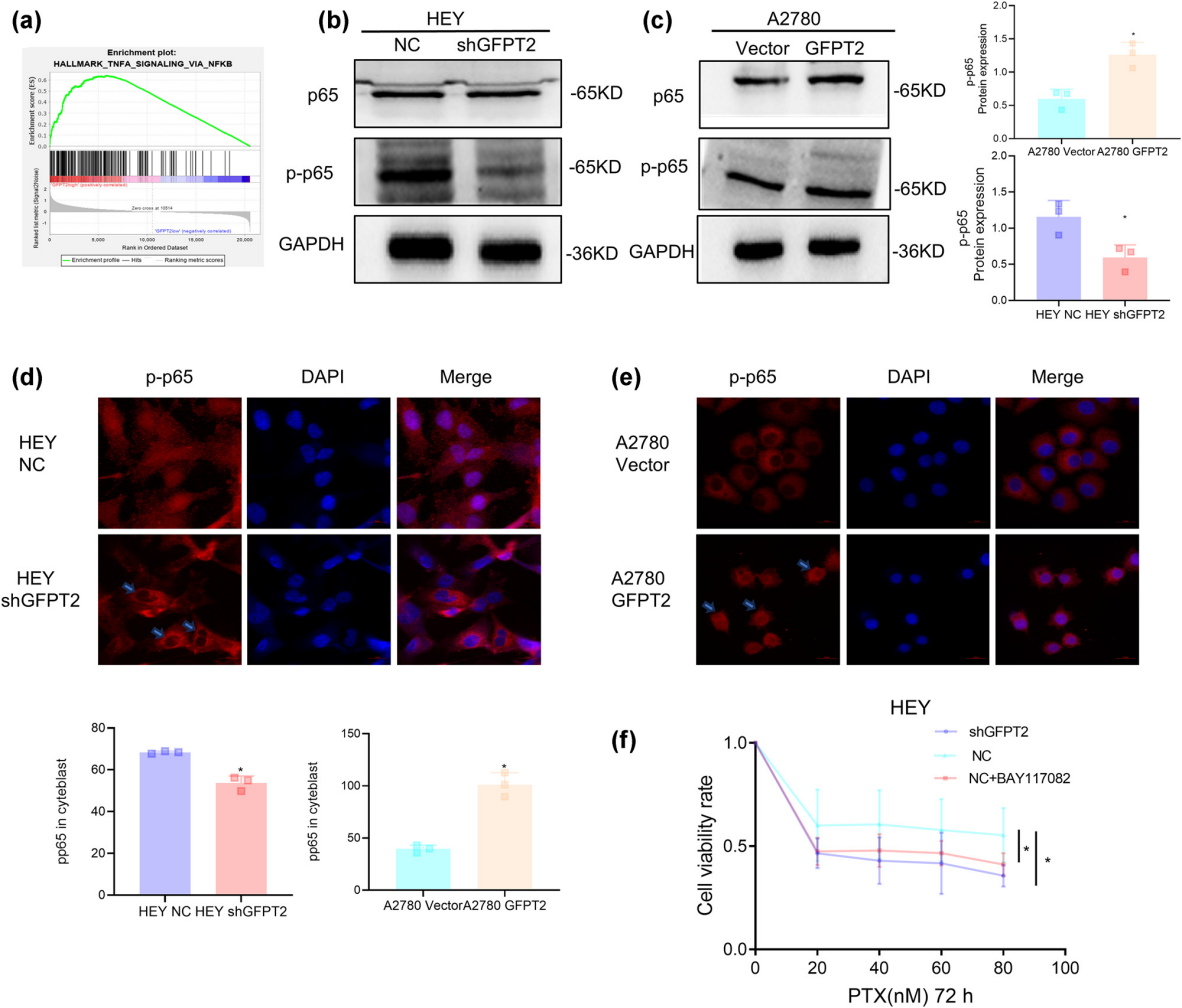
GFPT2 activates the NF-κB pathway in A2780 and HEY cells, (a) GSEA revealed DEGs between patients with high GFPT2 (*n* = 10) and low GFPT2 expression (*n* = 10) from TCGA, highlighting the upregulation of the NF-κB pathway by GFPT2 expression. (b) and (c) Expression of total p65 and phosphorylated p65 in HEY cells with GFPT2 knockdown (b) and A2780 cells with GFPT2 overexpression (c), with GAPDH as a control. (d) and (e) Subcellular localization of phosphorylated p65 (in red) in HEY and A2780 cells was visualized through immunofluorescence staining, with DAPI (in blue) indicating nuclear location. (f) HEY and A2780 cells were treated with indicated concentrations of paclitaxel and BAY11-7082, a suppressor of NF-κB activation, for 72 h, and cell viability was analyzed. Results are expressed as means ± standard deviation. *P < 0.05.

In our exploration of GFPT2’s mechanistic influence on the reaction of ovarian cancer cells to paclitaxel, we discovered a pivotal connection between GFPT2 expression and the NF-κB signaling pathway, which was reported to be a central modulator in cells’ responses to chemotherapeutic agents [7]. Figure 3a revealed the findings from a GSEA on TCGA ovarian cancer database, which highlighted an enrichment of genes within the NF-κB pathway that were differentially expressed between samples with high and low GFPT2 levels, hinting at GFPT2’s regulatory potential within this pathway (10 GFPT2 high‐expression samples and 10 GFPT2 low-expression samples). The examination of the HEY cell line following GFPT2 manipulation showed no change in total p65 levels, yet a marked reduction in the active, phosphorylated form of p65 upon GFPT2 knockdown (Figure 3b), suggesting GFPT2’s role in the pathway’s activation. In contrast, in the A2780 cell line, GFPT2 overexpression led to increased phosphorylated p65 levels (Figure 3c), indicating that GFPT2 could activate the NF-κB pathway, possibly by enhancing the active p65 subunit. Figure 3d and e, through immunofluorescence, underscored GFPT2’s influence on phosphorylated p65’s nuclear translocation. Decreased levels of GFPT2 result in reduced nuclear p65, while its overexpression promotes accumulation within the nucleus, underscoring the role of GFPT2 in the intracellular movement and activation of NF-κB. Lastly, Figure 3f depicts the impact of the NF-κB inhibitor BAY11-7082 on the HEY cell line’s sensitivity to paclitaxel, showing that BAY11-7082 enhances the cells’ responsiveness to paclitaxel to a degree comparable to the effect of reducing GFPT2 expression. This indicated that blocking NF-κB activity may counteract the paclitaxel resistance linked to elevated levels of GFPT2. These insights reveal the nuanced interplay between GFPT2 and the NF-κB pathway in regulating ovarian cancer cells’ sensitivity to paclitaxel, presenting the pathway’s modulation by GFPT2 as a promising avenue for therapeutic strategies in ovarian cancer treatment.

### 3.4 GFPT2 influences the expression level of NKX3-2

**Figure 4:**
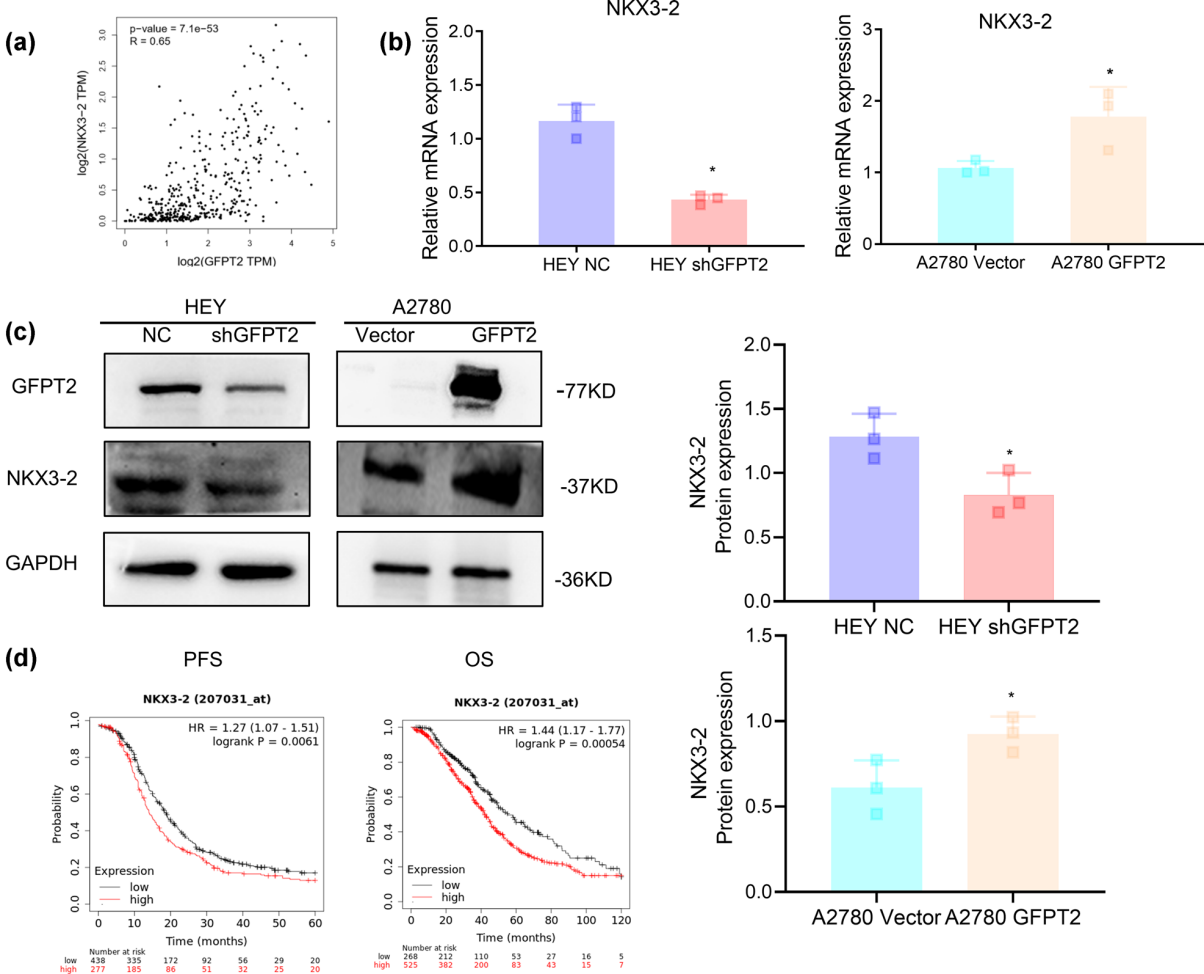
GFPT2 induces NKX3-2 expression in A2780 and HEY cells, (a) Correlation coefficient analysis of GFPT2 with NKX3-2 in ovarian cancer patients from TCGA database. (b) NKX3-2 mRNA expression, as measured by RT-PCR in HEY cells with GFPT2 knockdown and A2780 cells with GFPT2 overexpression, with GAPDH as a control. (c) NKX3-2 protein expression, as examined by Western blotting in HEY cells with GFPT2 knockdown and A2780 cells with GFPT2 overexpression, with GAPDH as a control. (d) Survival analysis of patients with ovarian cancer who received paclitaxel-containing chemotherapy, data derived from TCGA. Results are expressed as means ± standard deviation. *P < 0.05.

To further delineate the mechanisms by which GFPTT influences the NF-κB signal pathway, we thoroughly screened the top 100 differentially expressed genes (DEGs). This led to the identification of NKX3-2, which suggested a coordinated expression pattern between these two genes (http://gepia.cancer-pku.cn, Figure 4a) [8]. Earlier research indicates that NKX3-2 plays a role in activating the NF-κB signaling pathway [9,10]. Our subsequent investigations, detailed in Figure 4b and c, showed that manipulating GFPT2 levels by increasing or decreasing it correspondingly affected NKX3-2 at both the transcriptional and translational levels, suggesting a possible regulatory relationship. Additionally, Figure 4d, which includes survival data from TCGA database, suggested that higher *NKX3-2* expression in patients receiving paclitaxel treatment is linked to a worse prognosis, potentially making NKX3-2 a valuable prognostic marker for ovarian cancer. Taken together, these results indicate that GFPT2 influences the NF-κB pathway, at least partially, by regulating NKX3-2, providing fresh perspectives on the intricate signaling networks within ovarian cancer cells.

## 4 Discussion

Chemoresistance remains a significant therapeutic problem in the clinical management of EOC [11]. Paclitaxel-containing chemotherapy is critical in treating EOC, particularly for improving the prognosis of advanced patients [12]. However, most EOC patients initially respond to treatment but eventually develop recurrence and metastases and die of cancer within 5 years, owing primarily to chemoresistance.

Several well-established mechanisms contribute to the much-discussed resistance to paclitaxel, which is a standard anti-cancer drug [12]. These include the overexpression of the MDR-1 gene, modification of the target protein (β-tubulin), alteration of apoptosis-related proteins, and alteration of spindle checkpoint proteins [12,13]. Our study identified a potential connection between GFPT2 and paclitaxel resistance in EOC cells, where GFPT2 inhibits paclitaxel-induced apoptosis by activating the NF-κB pathway, possibly by influencing NKX3-2. Supporting this, TCGA database data show that elevated levels of both *GFPT2* and *NKX3-2* are associated with poor prognosis in EOC patients. Among the various proteins involved in O-GlcNAc synthesis, GFPT2 operates in the HBP [14]. O-GlcNAcylation, a type of epigenetic modification, is associated with the action of two enzymes, OGT and OGA, both subject to O-GlcNAcylation [15].

Caspases are critical players in the apoptotic process, and their activation is essential for starting and carrying out apoptosis [16]. Caspase-3 is particularly important among them, as its activation triggers the cleavage of various cellular components, leading to the distinct morphological and biochemical changes that define apoptotic cell death [16]. In our research, we found that overexpressing GFPT2 resulted in lower levels of cleaved Caspase-3 in A2780 cells that were treated with paclitaxel. This observation supports that GFPT2 may inhibit the activation of Caspase-3, diminishing the pro-apoptotic effects typically induced by paclitaxel [16]. This result aligns with the established role of Caspase-3 as a crucial mediator of apoptosis caused by chemotherapy, indicating that GFPT2 overexpression could interfere with the apoptotic process by affecting caspase activity.

Studies have shown that the NF-κB pathway can promote tumor resistance to apoptosis, which is a significant factor in developing chemoresistance in various cancers, including EOC [17,18], aligning with our findings that GFPT2 overexpression is associated with paclitaxel resistance in EOC cells through the activation of the NF-κB pathway. Once activated, NF-κB translocates to the nucleus and promotes the transcription of genes critical for cell survival and the regulation of apoptosis [19]. Therefore, the activation of NF-κB induced by GFPT2 may contribute to paclitaxel resistance by altering the expression of these genes. In addition, NF-κB interacts with other signaling pathways, including the mitogen-activated protein kinase (MAPK) pathway, which is essential for regulating cell survival during chemotherapy [20]. This interaction suggests a broader regulatory role in cellular responses to chemotherapy, potentially influencing the efficacy of paclitaxel treatment in EOC. Our findings show that GFPT2 overexpression results in heightened phosphorylation of p65, a critical subunit of NF-κB, and its subsequent nuclear translocation, thereby confirming the activation of the NF-κB pathway (Figure 3). This activation is associated with reduced apoptosis induced by paclitaxel, as evidenced by Caspase-3 expression (Figure 2), implying that GFPT2 may influence the sensitivity of EOC cells to paclitaxel. Further investigation is needed to understand how GFPT2 modulates paclitaxel resistance through this potential interaction between NF-κB and the MAPK pathways. Moreover, our study elucidates GFPT2’s influence on the NF-κB signaling pathway, highlighting its regulatory significance in the cellular response to paclitaxel treatment. This observation aligns with previous studies emphasizing NF-κB’s importance across various pathological conditions, including those arising from monosodium glutamate [21].

The NF-κB family of transcription factors comprises five members: RelA (p65), RelB, c-Rel, NF-κB1 (p50), and NF-κB2 (p52). Under resting conditions, NF-κB p65/p50 subunit dimers are sequestered in the cytoplasm, bound to inhibitory proteins [22]. Upon activation, these inhibitors undergo phosphorylation, ubiquitination, and subsequent proteasomal degradation, facilitating the release of NF-κB and allowing for the translocation of the p65–p50 complexes into the nucleus [23]. This cascade induces the transcription of target genes. The NF-κB pathway promotes cell proliferation, migration, invasion, and inhibiting apoptotic mechanisms [22,23]. Additionally, NF-κB acts as a pro-inflammatory transcription factor, significantly altering the expression of various genes associated with tumorigenesis [22,23]. Given that the treatment with paclitaxel elevated GFPT2 expression, it is plausible to infer that DNA damage and toxic responses triggered by paclitaxel would incite inflammatory reactions, subsequently activating the NF-κB pathway. This activation may spur the release of inflammatory factors and cytokines [22,23]. These events contribute to tumor initiation and chemoresistance, potentially leading to recurrence following chemotherapy [22,23]. GFPT2-induced O-GlcNAcylation has been previously noted to activate NF-κB signaling and establish a positive feedback loop in colorectal cancer [24,25,26]. Furthermore, both pathological and oncogenic O-GlcNAcylation influence NF-κB signaling through interactions with phosphorylation and acetylation [24,25,26]. Overall, our findings indicate that GFPT2 plays a pivotal role in modulating the NF-κB signaling pathway, which impacts paclitaxel resistance in ovarian cancer and highlights its broader implications in tumor progression and inflammatory responses.

Our research has potential clinical implications. GFPT2, as a regulator of the NF-κB pathway, presents another potential target for overcoming paclitaxel resistance. Inhibiting GFPT2 could disrupt the positive feedback loop with p65, thereby reducing the activation of NF-κB and enhancing the sensitivity of EOC cells to paclitaxel. Although specific GFPT2 inhibitors are not yet in clinical use, the understanding of their roles in cancer progression and resistance suggests that targeting GFPT2 could be a viable strategy in the future management of ovarian cancer. While there are no specific clinical trials targeting GFPT2 in the context of paclitaxel resistance in ovarian cancer, ongoing clinical trials explore the role of NF-κB inhibitors in various cancer treatments [27]. In summary, our study has unveiled an association of GFPT2 expression with the resistance of EOC cells to paclitaxel, probably through suppressing the pro-apoptotic effects of paclitaxel by activating the NF-κB pathway. Understanding the intricate relationships between GFPT2 and Caspases, proteins may offer new insights into the development of paclitaxel resistance in EOC and could inform the development of therapeutic strategies against this challenging malignancy.

To further address how GFPT2 regulates NF-κB activation, we identified NKX3-2 by screening the top DEGs. NKX3-2 is an evolutionarily conserved gene involved in embryonic development [28]. NKX3-2 is recognized for its ability to activate the NF-κB pathway by directly regulating the nuclear translocation of RelA, a vital component of the NF-κB complex [9]. Recent studies have underscored the role of NKX3-2 in ovarian cancer, demonstrating its potential to induce the migration of ovarian cancer cells [29]. Moreover, NKX3-2 expression is elevated in chemoresistant ovarian cancers as opposed to their chemosensitive counterparts, with high levels of NKX3-2 expression being significantly correlated with the occurrence of distant metastasis [30], which is consistent with our results from TCGA data analysis. Our findings suggest that NKX3-2 expression may be involved in activating the NF-κB pathway, which regulates genes related to cell survival, proliferation, and death. This implies that NKX3-2 could influence ovarian cancer cells’ susceptibility to paclitaxel. The potential link between NKX3-2 and drug resistance is supported by research indicating that *NKX3-2* is an EMT-related gene that may contribute to acquiring paclitaxel resistance in pancreatic carcinoma cells [31], suggesting its impact on chemotherapy response. In ovarian cancer cells, NKX3-2’s regulation of autophagy might affect the cells’ ability to withstand paclitaxel-induced stress [29], potentially aiding in cancer cell survival. In summary, our work demonstrates that GFPT2 expression is associated with resistance to paclitaxel in EOC cells, likely due to the attenuation of paclitaxel’s pro-apoptotic effects through NF-κB pathway activation. Understanding the complex interactions between GFPT2, NKX3-2, Caspases, and Bcl-2 family proteins may offer valuable insights into developing strategies to combat paclitaxel resistance in EOC, guiding the creation of novel therapeutic approaches for this challenging cancer.

While offering new insights into the role of GFPT2 in modifying paclitaxel resistance in ovarian cancer cells, this work has certain limitations. Our investigations indicate that the GFPT2 protein is upregulated in cells exposed to paclitaxel. Consequently, EOC cells are likely to evade the pro-apoptotic effects of paclitaxel by enhancing the activity of the NF-κB pathway. Exploring the underlying dynamics of GFPT2, Caspases, and the family of Bcl-2 proteins may be beneficial in understanding how EOC cells can resist paclitaxel, which antagonizes the directed therapeutics against these cells that have always been challenging. Our findings show that GFPT2 considerably impacts the NF-κB pathway and NKX3-2 levels, indicating a potential regulatory connection. However, the specific chemical processes by which GFPT2 affects NKX3-2 have yet to be determined. Further research is needed to understand the direct and indirect connections between these two proteins and the sequence of events that activate the NF-κB pathway under the impact of GFPT2. Furthermore, our gene enrichment analysis indicated the participation of other signaling pathways, such as the MAPK and WNT pathways, in the complex network of paclitaxel resistance. Their probable involvement in paclitaxel resistance shows that GFPT2 has a larger influence on cellular signaling. Future studies should go further into these pathways to better understand GFPT2’s influence on ovarian cancer cell biology and chemosensitivity. Furthermore, while our *in vitro* findings are encouraging, their translational significance must be validated by *in vivo* research.

## 5 Conclusion

**Figure 5:**
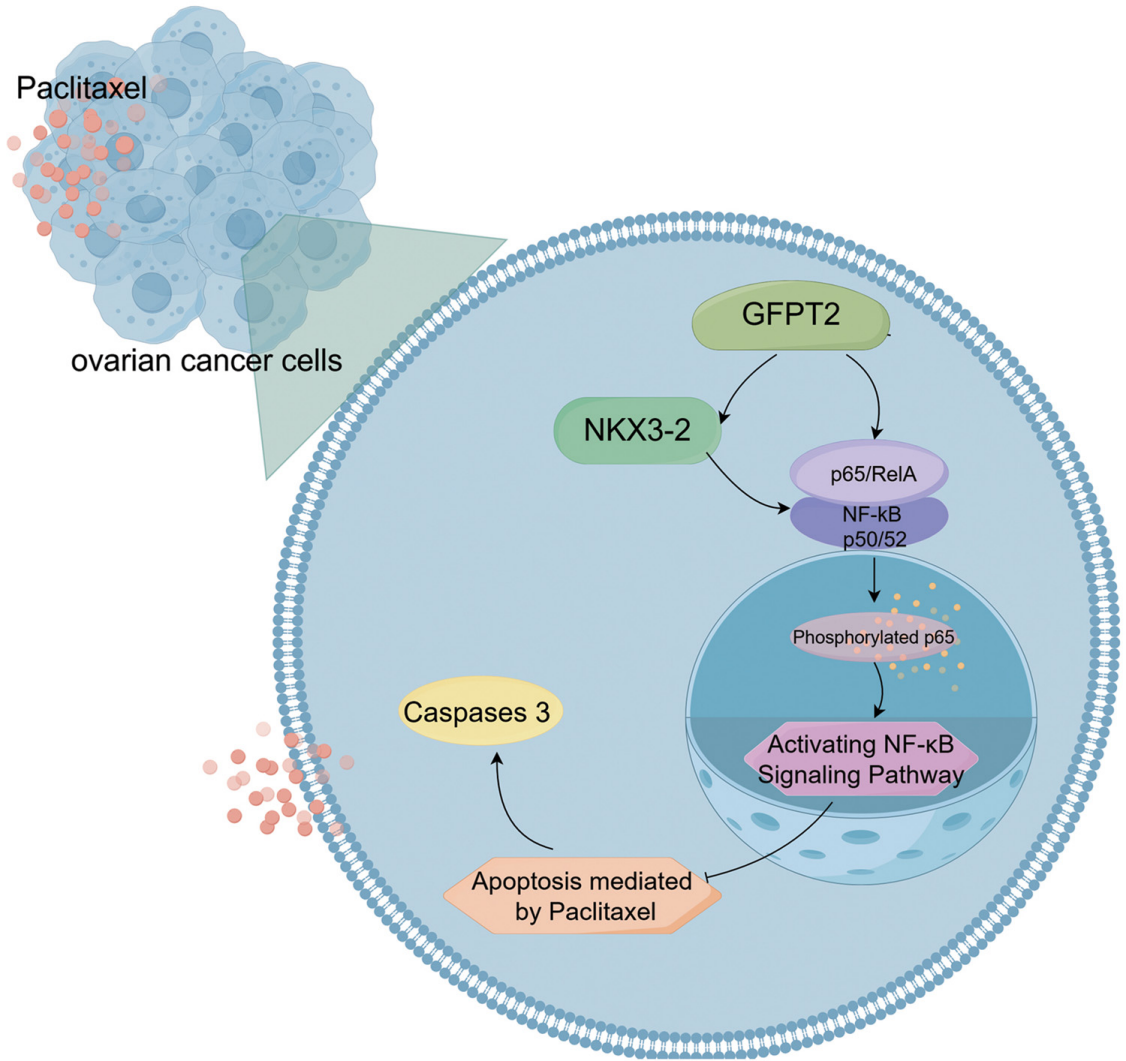
A schematic representation of the proposed mechanism by which GFPT2 influences paclitaxel resistance in EOC cells through the NF-κB pathway.

Our study has demonstrated a link between GFPT2 expression levels and the paclitaxel resistance of EOC cells. This resistance is possibly caused by the activation of the NF-κB pathway, which suppresses the pro-apoptotic effects of paclitaxel (Figure 5 plotted by Figdraw). These results may open the door for innovative treatment approaches to combat paclitaxel resistance in treating EOC.
